# Endothelial fibrinolytic response onto an evolving matrix of fibrin

**DOI:** 10.1186/s12878-016-0048-6

**Published:** 2016-04-14

**Authors:** O. Castillo, H. Rojas, Z. Domínguez, E. Anglés-Cano, R. Marchi

**Affiliations:** Centro de Medicina Experimental, Laboratorio Biología del Desarrollo de la Hemostasia, Instituto Venezolano de Investigaciones Científicas, Caracas, República Bolivariana de Venezuela; Universidad de Carabobo, Escuela de Bioanálisis (Sede Aragua), Maracay, República Bolivariana de Venezuela; Instituto de Inmunología, Universidad Central de Venezuela, Caracas, República Bolivariana de Venezuela; Laboratorio de Fisiología Celular, Centro de Biofísica y Bioquímica, Instituto Venezolano de Investigaciones Científicas, Caracas, República Bolivariana de Venezuela; Instituto de Medicina Experimental, Universidad Central de Venezuela, Caracas, República Bolivariana de Venezuela; Inserm UMR_S 1140, Faculté de Pharmacie, Paris, France; Université Paris Descartes, Sorbonne Paris Cité, Paris, France

**Keywords:** Fibrinogen, Fibrin, Fibrinolysis, Urokinase-type plasminogen activator, Plasminogen activator inhibitor type 1, Endothelium, Human dermal microvascular endothelial cells

## Abstract

**Background:**

Fibrin provides a temporary matrix at the site of vascular injury. The aims of the present work were (1) to follow fibrin formation and lysis onto the surface of human dermal microvascular endothelial cells (HMEC-1), and (2) to quantify the secretion of fibrinolytic components in the presence of fibrin.

**Methods:**

Fibrin clots at different fibrinogen concentrations were formed on top of (model 1) or beneath (model 2) the endothelial cells. Fibrin formation or lysis onto the surface of HMEC-1 cells, was followed by turbidity. Clot structure was visualized by laser scanning confocal microscopy (LSCM). The secretion of uPA and PAI-1 by HMEC-1 cells was quantified by ELISA.

**Results:**

The rate of fibrin formation increased approximately 1.5-fold at low fibrinogen content (0.5 and 1 mg/mL; *p* < 0.05) compared to the condition without cells; however, it was decreased at 2 mg/mL fibrinogen (*p* < 0.05) and no differences were found at higher fibrinogen concentrations (3 and 5 mg/mL). HMEC-1 retarded dissolution of clots formed onto their surface at 0.5 to 3 mg/mL fibrinogen (*p* < 0.05). Secretion of uPA was 13 × 10^−6^ ng/mL *per* cell in the absence of RGD and 8 × 10^−6^ ng/mL *per* cell in the presence of RGD, when clots were formed on the top of HMEC-1. However, the opposite was found when cells were grown over fibrin: 6 × 10^−6^ ng/mL *per* cell without RGD vs. 17 × 10^−6^ ng/mL *per* cell with RGD. The secretion of PAI-1 by HMEC-1 cells was unrelated to the presence of fibrin or RGD, 7 × 10^−6^ μg/mL *per* cell and 5 × 10^−6^ μg/mL *per* cell, for the apical (model 1) and basal clots (model 2), respectively.

**Conclusions:**

HMEC-1 cells influence fibrin formation and dissolution as a function of the fibrin content of clots. Clot degradation was accentuated at high fibrin concentrations. The secretion of fibrinolytic components by HMEC-1 cells seemed to be modulated by integrins that bind RGD ligands.

## Background

Fibrinogen is a 340 kDa plasma glycoprotein that circulates at approximately 2–4 mg/mL. The molecule is 45 nm long and comprises a symmetrical dimer consisting of two outer D domains and a central E domain, linked by α- helical coiled-coil rods. The fibrinogen molecule consists of two sets of three different polypeptide chains Aα, Bβ, and γ, joined by disulphide bonds at their N-termini E domain. The C-termini of the Bβ and γ chains are located in the D domain, while that of the Aα chains form a free non well-structured domain, the αC domain [1].

Fibrinogen does not polymerize spontaneously due to negative charges repulsion at the N-termini of the Aα- and Bβ- chains. The removal by thrombin of short peptide sequences at Aα A1-R16 (fibrinopeptide A, FpA) and Bβ Q1-R14 (fibrinopeptide B, FpB) triggers the association between the fibrin monomers (fibrinogen molecules devoid of FpA and FpB) [1, 2]. The release of fibrinopeptides A and B exposes the polymerization sites “A” (knob A) and “B” (knob B), which associate to constitutive polymerization sites located at the C-termini end of the γ chains (“a” or hole a) and β (“b” or hole b). The association of fibrin monomers give rise to protofibrils that have two monomer units of width. The lateral aggregation of protofibrils forms the fibrin fibers. It seems that FpBs release contribute to the lateral aggregation of protofibrils [3–5].

Coagulation and fibrinolysis are activated simultaneously in response to injury. The cross-linked fibrin is deposited in blood vessels and tissues, and plasmin is responsible of the soluble fibrin degradation products [6]. Under physiological conditions, activators, inhibitors, and cofactors finely regulated fibrinolysis. Plasmin is formed from its precursor (plasminogen, Pg) by tissue type plasminogen activator (tPA) at the surface of fibrin [7]. Furthermore, endothelial cells are involved in fibrinolysis regulation by secreting substances, such as tPA and plasminogen activator inhibitor type 1 (PAI-1). Although tPA is a poor plasminogen activator in solution, at the surface of fibrin the reaction is amplified approximately two orders of magnitude [7, 8].

Fibrin network structure depends on the quality and amount of fibrinogen, on thrombin and calcium concentrations, and on ionic strength, among others [9, 10]. The fibrin meshwork structure is characterized by fibrin thickness, fiber density, pores size, and rigidity that reflects fibrin FXIIIa cross-linking and clot structure [11]. It has been found that clots from patients with thrombotic disorders were composed by thin fibers with increased fibrin density and rigidity, and decreased clot lysis rate [12–14]. Endothelial cells (ECs) interact with fibrin at the sites of vascular injury, thrombosis, inflammation and tumour growth, whereas they are quiescent when exposed to circulating fibrinogen [15]. The regulation of fibrinolysis by the endothelium has been widely studied [16–19]; however, the response of ECs to different fibrin structures has not been explored as yet. This knowledge can be relevant in thrombotic or bleeding mechanisms.

In the present work, fibrin clots of different structure were prepared at varying fibrinogen concentrations. Fibrin polymerization and fibrinolysis onto the HMEC-1 surface were followed by turbidity. Furthermore, fibrin association to ECs receptors was visualized by LSCM in the presence and absence of the synthetic disintegrin RGD.

## Methods

### Materials

The MCDB 131 medium, foetal bovine serum, penicillin, streptomycin, fungizone, L-glutamine were purchased from GIBCO (Grand Island, NY, USA). Epidermal growth factor was from Invitrogen (Nalge Nunc International, Rochester, NY, USA). The 96 well microtiter plates and the 8 wells LabTek® Chamber Slide™ from Nalge Nunc International (Rochester, NY, USA). Lysine-sepharose was purchased from Health Care (Piscataway, NJ, USA). Bovine thrombin and RGD peptide were from Sigma (St Louis, MO. USA). The dyes di-8-anepps and Alexa 488 were from Molecular Probes (Eugene, OR, USA). FluoSpheres size kit # 1 carboxylate modified microspheres red fluorescent was from Molecular Probes (Eugene, OR, USA). The uPA and PAI-1 ELISA kits were purchased from American Diagnostica (Greenwich, Connecticut, USA).

### Endothelial cells culture

The human dermal microvascular endothelial cells (HMEC-1) were kindly donated by Dr. Edwin Ades, Department of Health and Human Services Centers for Disease Control and Prevention (CDC, Atlanta USA). The cells were cultivated with MCDB 131 medium supplemented with 10 % foetal bovine serum, penicillin 100 U/mL, streptomycin 100 μg/mL, L-glutamine 200 mM, and epidermal growth factor 10 ng/mL. For polymerization and fibrinolysis experiments, cells were plated in 96 wells microtiter plates, and for confocal microscopy experiments in 8 wells LabTek® Chamber Slide™. Cells were cultivated at 37 °C in a humid atmosphere with 5 % CO_2_ until ~80 % confluence. The optimal quantity of thrombin to be used in order to clot fibrinogen without affecting cell morphology and viability was standardized. After cells reached ~80 % confluence, 1 to 5 nM thrombin was incubated on the surface of the cells monolayer during 2 h. The cells’ morphology was observed with an optical microscope (unchanged at 1 nM; results not shown). Similarly, the number of cells to be seeded in the microplate wells and confocal microscopy chambers were previously standardized in order to obtain 80 % confluence overnight.

### Fibrinogen purification

Fibrinogen was purified from pooled human plasma obtained from healthy donors. The Ethics Committee of the Instituto Venezolano de Investígaciones Científicas (IVIC) approved the project, and all subjects signed an informed consent before blood withdrawal. Blood was collected in citrate (1 volume of 0.13 M trisodium citrate and 9 volumes of blood). Immediately centrifuged at 2500 × g during 20 min, at 4 °C. Fibrinogen was precipitated by salting out using β-alanine, essentially as described elsewhere [20]. The plasminogen was removed from fibrinogen preparation using a lysine-sepharose column, following the manufacturer’s instructions. The clottability of the purified fibrinogen was >90 %.

### Fibrin polymerization and fibrinolysis on HMEC-1

The HMEC-1 cells (100,000 cells/well) were seeded on 96-well microtiter plate and cultivated overnight. The following day the medium was discarded and the fibrin meshwork was formed on top of the cells. Clots directly formed on the plastic surface of the microtiter plate served as controls. In an Eppendorf tube 143 μL of purified fibrinogen (0.5–5 mg/mL, final), 40 μL MCDB 131 non-supplemented medium and 17.5 μL of bovine thrombin – CaCl_2_ (1 nM and 2 mM, respectively, final) were mixed and immediately transferred on the top of the cells or directly on the plastic well surface. The optical density (OD) was read every 2 min during 100 min at 350 nm in an Infinite 200 M (Tecan, Vienna, Austria). For each curve it was calculated the slope (mOD/s)×100 and the maximum absorbance (MaxAbs, mOD). Experiments were run at least three times by triplicate. Fibrinolysis was triggered by the addition of 100 nM Pg and 0.145 nM tPA (previously standardized) to fibrinogen solutions (0.5–3 mg/mL) before clotting with thrombin – CaCl_2_. The OD was recorded every minute until the OD reached baseline values. For each curve it was measured the time needed to decrease by 50 % the maximum absorbance (T50; s), the lysis rate, in the descending part of the curve (LR; mOD/s)×100; and the area under the curve (AUC; mOD × s). Experiments were run at least three times by duplicate.

At the end of each experiment the morphology of the cells was checked with an optical microscope.

### Fibrin interaction with HMEC-1

Cells (120,000) were seeded in LabTek glass chamber slide and maintained at 37 °C in a humid atmosphere with 95 % air and 5 % CO_2_ and grown-up to 80 % confluence. The culture medium was removed and cells were labelled with 4 μM di-8-anepps for 15 min. The cells were then washed three times with phosphate buffered-saline (PBS). Fibrinogen (0.5, 2, and 5 mg/mL) mixed with 1 mM RGD (or the equivalent volume of buffer) and Alexa Fluor 488-labeled fibrinogen (19 μg/206 μL sample volume), was clotted with 1 nM thrombin – 2 mM CaCl_2_ (final). The clotting mixture was immediately transferred over the cells or directly to the bottom of the glass chamber (control, without cells). Clot formation was allowed to progress for 2 h in a tissue culture incubator. Finally, the clot’s surface was covered with supplemented medium without serum. Duplicates of each condition were performed at least in three independent experiments.

In order to discard that the interaction of fibrin with the cells was merely an adsorption phenomenon, 2 μm fluorescent microspheres were included in the reaction mixture before adding thrombin.

The clot structure on the surface of HMEC-1 was visualized using a Nikon Eclipse TE 2000-U laser microscope (with a 488 nm Argon or 543 nm HeNe laser). The objective used was Plan Apo VC 60X in water immersion with a work distance of 0.27. The acquisition pinhole was set to 60 μm. For each clot several fields were examined at random before digital recording. Five areas of 212 × 212 μm (x,y) were selected from each duplicate. A Z- stack was imaged from the bottom of the dish (0 μm) to different distances from the surface of the cells, with step sizes of 0.5 μm. The fibers diameter and density were measured from the volume render obtained with the Olympus FV10-ASW 2.1, and peak analysis from OriginPro 8.

### Urokinase-type plasminogen activator (uPA) and plasminogen activator inhibitor 1 (PAI-1) secretion by HMEC-1 in the presence of fibrin

Fibrinogen was polimerized on the top of a HMEC-1 culture (model 1) or the cells were grown on fibrin as substratum (model 2). In model 1, fibrin was formed on HMEC-1 cells (100,000) using different fibrinogen concentration (0.25, 0.5, 1, 1.5, 2, 3, and 5 mg/mL). Clots were allowed to form during 30 min in the incubator and 200 μL of medium without serum was then added. The supernatant was carefully collected after 12 h and stored at −80 °C until use. The following protocol was used with RGD: a cell monolayer was incubated during 3 h with 1 mM RGD prepared in supplemented MCDB 131 medium. The medium was then discarded and the cells washed with PBS. A fibrin reaction mixture with 1 mM RGD was added on the top of the cells. The basal condition consisted of the cell monolayer incubated during 12 h with supplemented MCDB 131 medium.

In order to cultivate the HMEC-1 on a fibrin layer (model 2), thin fibrin films of 30 μL were formed on the bottom of the 96-well microplate, using the same clotting condition as model 1. After 30 min at 37 °C, 100,000 cells/well supplemented or not with 1 mM RGD were seeded on the top of the fibrin and the plate left overnight in the incubator. The following day fresh medium without serum was added, and incubated for 12 h. The supernatant was collected and kept at −80 °C until use. Experiments were performed in triplicate and uPA and PAI-1 concentrations were measured in the cell supernatant by ELISA. The basal condition consisted of the cells monolayer incubated during 12 h with supplemented MCDB 131 medium.

The quantity of uPA and PAI-1 secreted was normalized to the number of cells *per* well.

### Statistical analysis

Statistical analysis was performed with OriginPro version 8.1. Descriptive statistics: mean, standard deviation (SD) or the standard error of the mean (SEM) were calculated. Normality was assessed by Shapiro-Wilk Test. Means were compared by one-way-ANOVA. A significance level of 0.05 was used.

## Results

### Fibrin polymerization and fibrinolysis on the top of HMEC-1

**Fig. 1:**
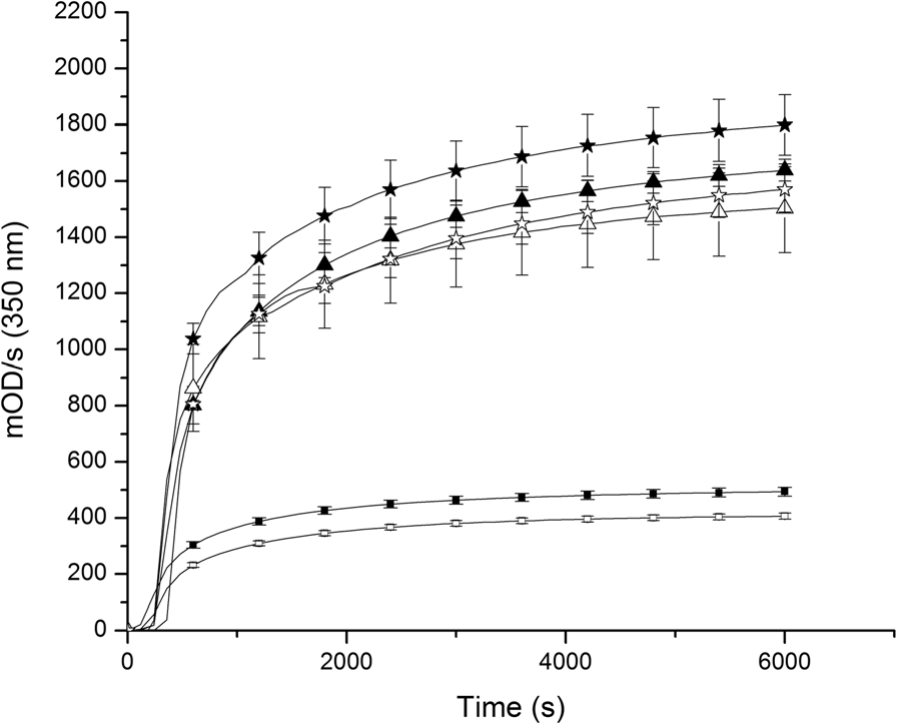
Fibrin polymerization on the top of HMEC-1 at different fibrinogen concentrations. Filled symbols represent the condition of fibrin formed on the top of the cells and empty symbols clots formed directly on the plastic dish. (■, □): 1 mg/mL, (▲, △): 3 mg/mL and (★, ☆): 5 mg/mL

**Fig. 2:**
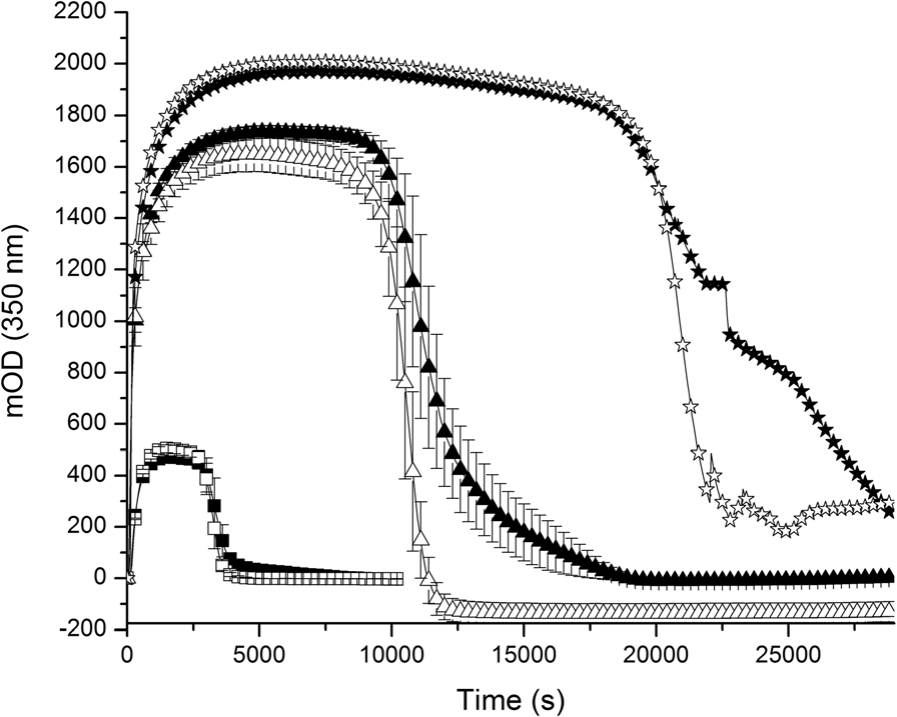
Fibrin degradation on the top of HMEC-1 at different fibrinogen concentrations followed by turbidity. (■, □): 1 mg/mL, (▲, △): 2 mg/mL and (★, ☆): 3 mg/mL. Filled symbols: fibrin formed on the top of the cells; empty symbols: clots formed directly on the plastic dish

The slope and MaxAbs increase steadily from 0.5 to 5 mg/mL both when fibrin was formed on the top of HMEC monolayer or without cells (Table 1). Figure 1 shows the time course of fibrin formation at 3 different fibrinogen concentrations (1, 3 and 5 mg/mL). The influence of fibrinogen concentration on the kinetics of fibrin polymerization is clearly evidenced. In the presence of cells MaxAbs was higher compared to the condition without cells. Fibrinolysis results are summarized in Table 2. The lysis rate (LR) was slightly but significantly decreased in the presence of cells at the fibrinogen concentrations tested (0.5 to 3 mg/mL). However, the time needed for 50 % of clot lysis (T50%) was similar. In Fig. 2 are shown the time course of fibrinolysis at 1, 2, and 3 mg/mL fibrinogen.

**Table 1:** Summary of the kinetics of fibrin polymerization on the top of HMEC-1 at different fibrinogen concentrations

	Fibrin + Cells		Fibrin	
Fg (mg/mL)	Slope (mOD/s)×100	MaxAbs (mOD)	Slope (mOD/s)×100	MaxAbs (mOD)
0.5	50 ± 7*	305 ± 20*	33 ± 4	221 ± 14
1	83 ± 3*	494 ± 16*	61 ± 2	406 ± 10
2	111 ± 5*	976 ± 45	132 ± 11	1019 ± 58
3	519 ± 59	1715 ± 42	668 ± 154	1543 ± 179
5	710 ± 37	1890 ± 113*	710 ± 103	1657 ± 82

Results are expressed as the mean (± SD)

*Fg* fibrinogen, *MaxAbs* maximum absorbance

*p < 0.05 Comparison between fibrin polymerization parameters of clots formed on the top of the cells with those performed in its absence

**Table 2:** Summary of the fibrin degradation on the top of HMEC-1

	Fibrin + Cells			Fibrin		
Fg (mg/mL)	T50% (s)	LR (mOD/s) ×100	AUC (mODxs) ×10^6^	T50% (s)	LR (mOD/s) ×100	AUC (mODxs) ×10^6^
0.5	870 ± 79	41 ± 4*	0.3230 ± 0.0204	820 ± 17	48 ± 2	0.2946 ± 0.0147
1	1790 ± 173	58 ± 0.8*	1.5004 ± 0.1617	1650 ± 60	77 ± 2	1.4120 ± 0.0843
2	4330 ± 466	54 ± 2.3*	7.5957 ± 0.0927*	4150 ± 69	68 ± 1.4	6.8737 ± 0.2645
3	5860 ± 259	77 ± 21*	19.6071 ± 1.0103*	5690 ± 193	135 ± 1.4	16.1075 ± 1.0519

Results are expressed as the mean (± SD)

*Fg* fibrinogen, *T50%* time required to decrease the MaxAbs 50 %, *LR* lysis rate, *AUC* area under the curve

*p < 0.05 Comparison between fibrin degradation parameters of clots formed on the top of the cells with those performed in its absence

### Fibrin interaction with HMEC-1

**Fig. 3:**
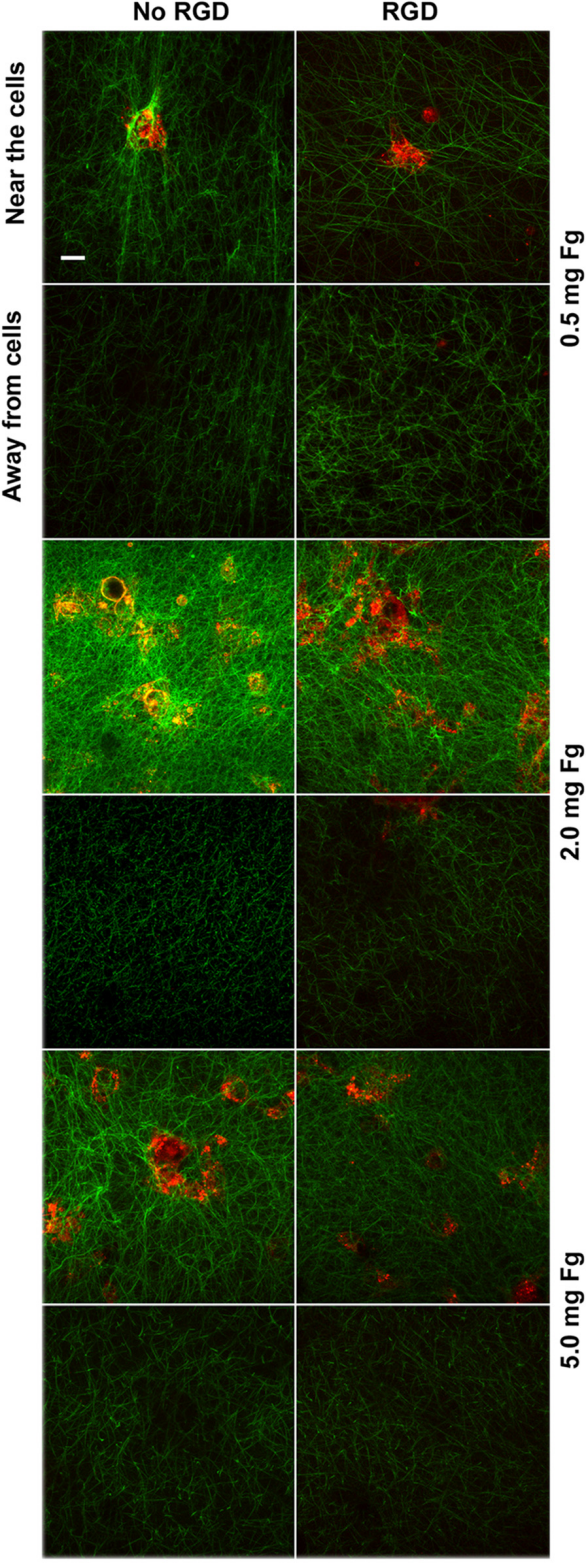
Laser scanning confocal microscopy images of clots formed on the top of HMEC-1 at different fibrinogen concentrations. The fibrin fibers were visualized with Alexa 488 and the cell membrane with di-8-anepps. The pictures show the fibrin fibers arrangements of clots supplemented or not with 1 mM RGD near the cell surface at 0.5, 2 and 5 mg/mL fibrinogen, and at 15 μm away. The tool bar represents 20 μm

Fibrin network formed at three different fibrinogen concentrations (0.5, 2, and 5 mg/mL) on the top of HMEC-1 monolayers were digitized near the cell surface and at 15 μm, both in the presence and absence of 1 mM of the synthetic peptide RGD that competes with fibrinogen for integrin-ligand binding. In Fig. 3 it is clearly seen that at 0.5 and 2 mg/mL the fibrin fibers interacted profoundly with the cell surface, the fibers looked radially stressed and the colocalization (in yellow) of the fibrin (green) with the cells membrane (red) is evidenced. However, at 5 mg/mL the interaction with the cell surface was rather decreased. This peculiar fibrin fibers distribution disappears with distance from the cell surface. At approximately 15 μm, the fibers looked uniformly distributed. When RGD was added to the fibrinogen solutions, the interaction between fibrin and cells decreased.

**Fig. 4:**
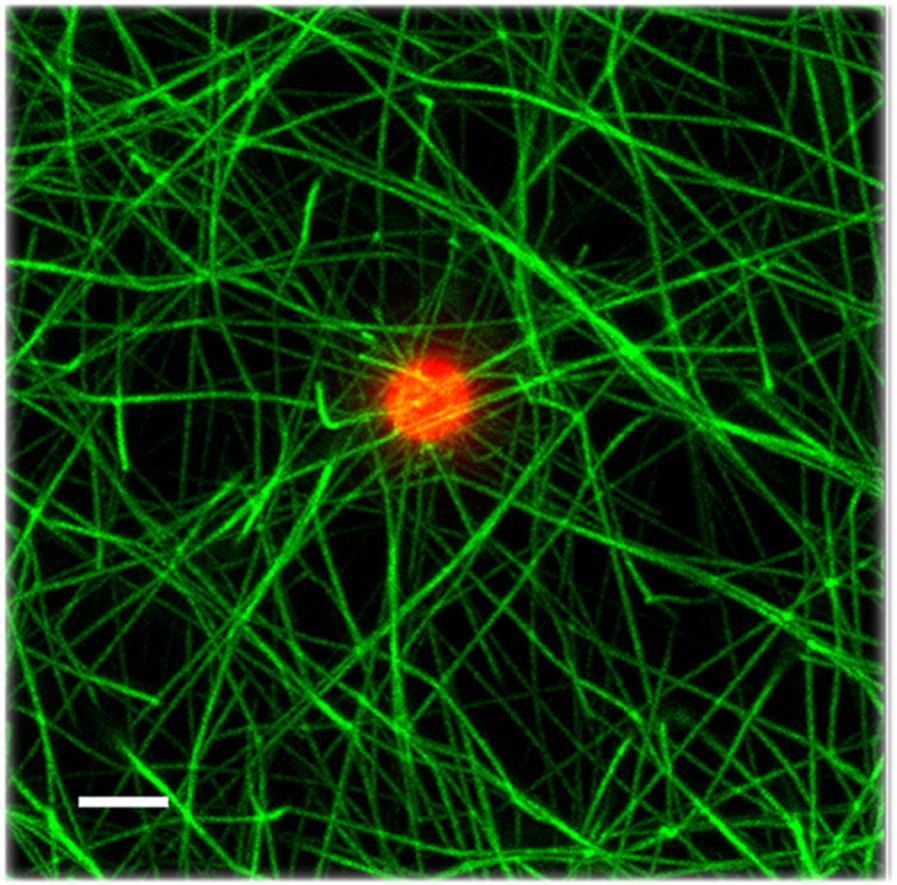
Fibrin network formed with fluorescent microspheres. A field with only one bead was magnified in order to appreciate that these particles did not interact with fibers. The tool bar represents 2 μm

In order to rule out that the fibrin association to the cells was merely an adsorption phenomenon, fluorescent microspheres of 2 μm were incorporated into the clotting mixture. The fibrin fibers did not interact with the beads nor looked stressed, confirming that fibrin fibers are interacting with specific receptors on the cell membrane (Fig. 4).

The fibrin network was characterized by measuring fibrin fiber diameter and fibrin density near the cell surface and at 15 μm (Table 3). Fibrin fibers were thicker near the cell surface compared to that observed at 15 μm at all fibrinogen concentrations tested without RGD. In contrast, in the presence of RGD there was not such relationship. Apparently, only at 5 mg/mL the fibrin fibers diameter did not change. Fibrin density was greater near the cell surface both in the presence or absence of RGD, except at 0.5 mg/mL in the presence of RGD (*p* > 0.05).

**Table 3:** Characterization of fibrin networks formed on the surface of HMEC-1 culture by laser scanning confocal microscopy (LSCM)

Fibers diameter (μm)				
Fg (mg/mL)	- RGD Near	- RGD Far	+ RGD Near	+ RGD Far
0.5	2.02 ± 0.05*	1.65 ± 0.05	1.49 ± 0.09*	1.72 ± 0.07
2	1.98 ± 0.12*	1.76 ± 0.12	1.99 ± 0.06*	1.62 ± 0.07
5	2.03 ± 0.65*	1.52 ± 0.33	2.00 ± 0.10	1.95 ± 0.33
Fibrin density (peaks/μm)				
Fg (mg/mL)	- RGD Near	- RGD Far	+ RGD Near	+ RGD Far
0.5	0.192 ± 0.052*	0.099 ± 0.045	0.205 ± 0.051	0.160 ± 0.054
2	0.299 ± 0.029*	0.135 ± 0.035	0.270 ± 0.023*	0.152 ± 0.030
5	0.254 ± 0.030*	0.141 ± 0.057	0.282 ± 0.022*	0.138 ± 0.056

Fibrin fibers diameter and density were quantified from LSCM images Values are expressed as mean (± SEM) of 3 experiments performed in duplicate

*p < 0.05. Comparisons are between near the cells surface vs. far, without (−) and with (+) RGD

**Fig. 5:**
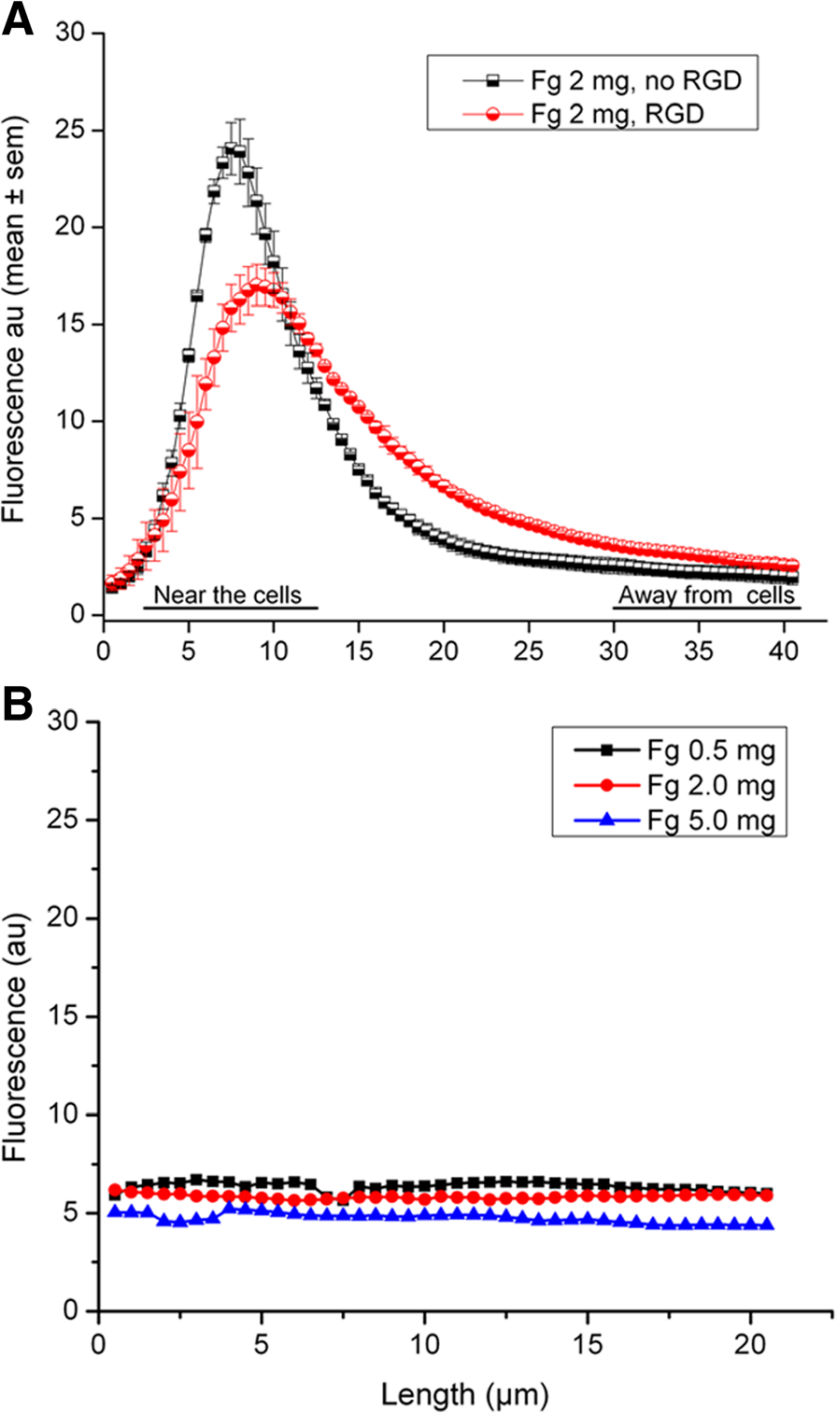
Representation of the changes of the mean fluorescence intensity according to the distance from the bottom of the dish up to 40 μm. **a** Without and with RGD at 2 mg/mL fibrinogen. **b** Without cells at 0.5, 2 and 5 mg/mL fibrinogen

Changes in fluorescence intensity as a function of the distance from the cell surface at 2 mg/mL of fibrinogen is shown in Fig. 5.

### HMEC-1 secretion of uPA and PAI-1 in the presence of fibrin

The basal secretion of uPA was 7.0 ± 0.5×10^−6^ ng/mL *per* cell and that of PAI-1 6.7 ± 0.5×10^−6^ μg/mL *per* cell. Since the secretion of uPA and PAI-1 was almost similar at the different fibrinogen concentrations tested in the presence or not of RGD, these values were averaged and reported in Table 4. In model 1, when fibrin was formed on the top of the cells monolayer, uPA secretion in the absence of RGD was ~2-fold higher than in its presence (*p* < 0.05). However, the opposite was found for model 2. When cells were grown on the top of the fibrin network the secretion of uPA in the absence of RGD was decreased 2-fold (*p* < 0.05). The secretion of PAI-1 in model 1 was 1.3-fold higher compared to model 2 (*p* < 0.05). The PAI-1 concentration in the presence of fibrin was comprised in the 95 % CI of HMEC-1 basal secretion.

**Table 4:** HMEC-1 cells secrete urokinase-type plasminogen activator and plasminogen activator inhibitor type 1

μg/mL *per* cell	Model 1	Model 2
uPA×10^−9^ (−RGD)	12.7^*,**^ (11.2–14.1)	6.2^*^ (5.0–7.4)
uPA×10^−9^ (+RGD)	7.5^**^ (5.9–9.1)	17.3 (15.0–19.6)
PAI-1×10^−6^ (−RGD)	7.87^**^ (6.65–9.10)	5.97 (4.50–7.44)
PAI-1×10^−6^ (+RGD)	7.38^**^ (6.54–8.23)	5.08 (3.01–7.16)

Urokinase and PAI-1 were quantified by ELISA in HMEC conditioned-medium obtained in the presence of fibrin. Fibrin was formed on the top of the cells monolayer (model 1) or cells were seeded on the top of fibrin film (model 2). The basal secretion of uPA was 7.0 ± 0.5 × 10^−9^ μg/mL *per* cell, and that of PAI-1 6.7 ± 0.5 × 10^−6^ μg/mL *per* cell. The quantity of uPA and PAI-1 secreted was normalized to the number of cells. Results are expressed as the mean and the 95 % confidence interval (CI) in brackets

*p < 0.05 uPA secretion without RGD (−RGD) compared to that with RGD (+RGD) for model 1

**p < 0.05 uPA secretion without RGD of model 1 compared to that of model 2, and with RGD in model 1 compared to that in model 2

## Discussion

The aims of the present work were (1) to study fibrin formation and lysis onto the surface of HMEC-1 cells, and (2) to quantify uPA and PAI-1 secretion in the presence of fibrin. We also analyzed the fibrin meshwork formed on the top of HMEC-1 cells by laser scanning confocal microscopy. The structure of fibrin was modulated by modifying the concentration of fibrinogen.

Fibrin is pro inflammatory, pro-angiogenic and may contribute to the development of inflammatory disease processes and tumorigenesis [21–23]. Atherogenesis is a multifactorial process and the atherosclerotic lesions may be initiated by the interaction of fibrin with the endothelium [24]. In normal physiologic conditions, the endothelium displays antiplatelet, anticoagulant and fibrinolytic properties [25].

In order to measure the secretion of fibrinolytic components from HMEC-1 we have used an adhesion model, where HMEC-1 cells were seeded directly on the plastic surface and after reaching 80 % of confluence fibrin was formed on their top (model 1) or cells were grown on a fibrin film (model 2).

The type of plasminogen activator secreted by HMEC-1 has been differently reported in the literature [26–28]. However, a majority of reports [29–31], including those from the group of Angles-Cano [26, 32, 33], have clearly demonstrated, using methods that identify either the molecular mass (Western blot) or the activity of the molecular mass (fibrin or casein zymography) that only uPA (54 kDa) is detected in culture media or lysates from HMEC-1. We have verified that the HMEC-1 line used in these studies secretes uPA (lysis band at 54 kDa by zymography).

Similar to what has been found for other ECs cultured in vitro, HMEC-1 basal PAI-1 secretion was higher than uPA, approximately 1000-fold [34–36]. In model 1, without RGD, the amount of uPA secreted to the medium was 2-fold higher compared to the basal secretion and to model 2. This difference was attributed to the availability of thrombin. In model 1, at the beginning of fibrin formation, thrombin is in solution and directly in contact with the cells, while in model 2 fibrin was already gelled, and less thrombin was accessible to the cells. The response of the ECs to thrombin depends on their origin, in human umbilical vein endothelial cells (HUVEC) the quantity of tPA and PAI-1 is dose-dependent on thrombin concentration, but not in human omental tissue microvascular endothelial cells (HOTMC) [35].

During fibrin formation from 0.5, 1, and 5 mg/mL fibrinogen the OD increased faster in the presence of HMEC-1 than without it (*p* < 0.05). Tietze et al. found similar results with human mesothelial cells (HOMC) but at constant fibrinogen concentration [34]. This “catalytic” cell effect at low fibrinogen concentration may be an advantage for patients with hypofibrinogenemia, which could contribute to stop faster blood extravasation. The rate of fibrin degradation in the presence of HMEC-1 was significantly decreased at all fibrin concentration (0.5 to 3 mg/mL), reaching a maximum value at 3 mg/mL (2-fold less). These results were attributed to several factors. The presence of PAI-1 (basal secretion ~ 6.7×10^−6^ μg/mL *per* cell) could decrease both the functional availability of uPA and tPA (externally added tPA), although the tPA bound to fibrin is less susceptible to PAI-1 inactivation [7, 37]. The peculiar fibrin structure observed near the cell surface could impair clot dissolution. In general, fibrin network with increased fibers density are digested slower [38]. However, opposite results were found with HOMC, where the T50% was decreased in the presence of cells [34]. The results reported by these authors were intriguing, since clots formed without cells were totally degraded earlier than those in the presence of cells.

It was previously found that fibrin fibers forms clumps near the surface of HUVEC culture; however, at 50 μm this pattern disappear and they distributed homogeneously [39]. The integrin involved in this interaction was the αvβ_3_ [39]. This integrin binds the ligand at specific RGD sequences of different adhesive proteins such as fibronectin, vitronectin, fibrinogen, among others [40]. In the present work the fibrin network structure formed at different fibrinogen concentrations was analyzed by LSCM. As expected, increasing the fibrinogen concentration increases the fibrin density [41], and the fibers associated to the cell surface looked stressed (Fig. 3), as has already been observed in other works at a given fibrinogen concentration [39, 42, 43]. Interestingly, at high fibrinogen concentration (5 mg/mL) the interaction of fibrin with the cells was decreased. Indeed, at increasing fibrin content, the number of protofibrils *per* fiber are greater [44], probably diminishing the accessibility of the RGD sites on the fibrin fibers (Aα 572–575) to integrins [40, 45].

The changes in fibrin fiber diameter at varying fibrinogen concentrations were not clearly appreciated by LSCM in spite of the low thrombin concentration used. This is probably due to the lower resolution limit of LSCM (~200–400 nm).

## Conclusions

We demonstrated that HMEC-1 influenced fibrin formation as a function of the fibrinogen input. However, the rate of fibrin degradation in the presence of HMEC-1 was significantly decreased at all fibrin concentrations. Impairment of fibrin binding to its cell receptor by RGD influenced the secretion of fibrinolytic components thus suggesting a role for fibrin binding in this mechanism. The next step would be to investigate the signalling pathway involved in the αvβ_3_ integrin activation, probably coupled to thrombin. A limitation of our study is that the results were obtained using HMEC-1 cells and may therefore be pertinent only to this cell line.

## Abbreviations

ECs: endothelial cells
ELISA: enzyme-linked immunosorbent assay
Fg: fibrinogen
FXIIIa: activated factor XIII
HMEC: human dermal microvascular endothelial cells
HOMC: human mesothelial cells
HOTMC: human omental tissue microvascular endothelial cells
HUVEC: human umbilical vein endothelial cell
LR: lysis rate
LSCM: laser scanning confocal microscopy
MaxAbs: maximum absorbance
OD: optical density
PAI-1: plasminogen activator inhibitor type 1
PBS: phosphate buffered-saline
Pg: plasminogen
RGD: arginyl glycyl aspartic acid
SEM: standard error of the mean
tPA: tissue type plasminogen activator
uPA: urokinase-type plasminogen activator
